# Supplementary material for: Synchronization of phase oscillators with frequency-weighted coupling
Source: Sci Rep. 2016 Feb 23;6:21926. doi: 10.1038/srep21926 (PMC4763290; doi:10.1038/srep21926)
Supplement: Supplementary Information [file srep21926-s1.pdf]

Supplementary Material. Synchronization of phase oscillators with frequency-weighted coupling

Can Xu,[1,2] Yuting Sun,[2] Jian Gao,[2] Tian Qiu,[3] Zhigang Zheng,[1,*] and Shuguang Guan[3,†]

[1]*College of Information Science and Engineering, Huaqiao University, Xiamen 361021,China*
[2]*Department of Physics and the Beijing-Hong Kong-Singapore Joint Centre for Nonlinear and Complex Systems (Beijing), Beijing Normal University, Beijing 100875, China*
[3]*Department of Physics, East China Normal University, Shanghai 200241, China*

The proof of empty discrete spectrum when K is sufficiently small. The key point to prove this is to demonstrate that the left side of Eq. (30) (in the main text) is bounded for any real x, y. Actually, the left side of Eq. (30) (in the main text) is less than

$$\int_{-\infty}^{1} \frac{x|\omega|g(\omega)}{x^2+(\omega-y)^2}d\omega + \int_{1}^{\infty} \frac{x|\omega|g(\omega)}{x^2+(\omega-y)^2}d\omega + \int_{-1}^{1} \frac{xg(\omega)}{x^2+(\omega-y)^2}d\omega. \tag{1}$$

It has been proved that the last term in (1) is bounded for any real values x and y [34]. Now we only need to prove the boundedness of the first and second term in (1). We notice that the second term of (1) can be rewritten as

$$\int_{1}^{y-\delta} \frac{x\omega g(\omega)}{x^2+(\omega-y)^2}d\omega + \int_{y+\delta}^{\infty} \frac{x\omega g(\omega)}{x^2+(\omega-y)^2}d\omega + \int_{y-\delta}^{y+\delta} \frac{x\omega g(\omega)}{x^2+(\omega-y)^2}d\omega, \tag{2}$$

provided that $y > 1$ and $\delta > 0$ is small. Evidently, the first and second terms in (2) are also bounded since there is no singularity in the finite integrating range. In addition, the third term in (2) can be calculated through integration by parts as

$$\frac{x\omega}{x^2+(\omega-y)^2}G(\omega)\Big|_{y-\delta}^{y+\delta} + \int_{y-\delta}^{y+\delta} \frac{x(\omega^2-x^2-y^2)}{[x^2+(\omega-y)^2]^2}G(\omega)d\omega, \tag{3}$$

where $G(\omega)$ is the primitive function of distribution density $g(\omega)$, i.e., $G(\omega)$ is continuous, $G(-\infty) = 0$ and $G(+\infty) = 1$. Thus the first term of (3) is bounded, and the second term of which can be reduced as

$$\int_{0}^{\delta} \frac{x(\omega^2-x^2)}{(x^2+\omega^2)^2}\left[G(y+\omega)+G(y-\omega)\right]d\omega + \int_{-\delta}^{\delta} \frac{2x\omega y}{(x^2+\omega^2)^2}G(\omega+y)d\omega. \tag{4}$$

The last term of (4) is also bounded and the first term in (4) could be estimated by the mean value theorem of integral as

$$2G(y)\left(-\frac{x\xi}{x^2+\xi^2}\right) + \left[G(y+\delta)+G(y-\delta)\right]\left(\frac{x\xi}{x^2+\delta^2} - \frac{x\delta}{x^2+\delta^2}\right), \tag{5}$$

where $0 \le \xi \le \delta$. Considering the property of $G(\omega)$, (5) is also bounded for any real x and y. Therefore, the second term in (1) is bounded. Similarly, the same strategy could also be adopted to prove the boundedness of the first term in (1).

*Electronic address: `zgzheng@hqu.edu.cn`
†Electronic address: `guanshuguang@hotmail.com`

The center manifold calculation for the bifurcation of the incoherent state. Based on the center manifold reduction, Ref. [37] provided a paradigmatic framework to obtain the amplitude equations near the critical point in classical Kuramoto model. Following this framework, we find that the frequency-weight Kuramoto model can also be treated. Considering Eqs. (34)-(37) in the main text, the important feature for the perturbed equation is its symmetry. The group $O(2)$ is generated by the rotations

$$\phi \cdot (\theta, \omega) = (\theta - \phi, \omega), \tag{6}$$

and the reflection

$$\kappa \cdot (\theta, \omega) = (-\theta, -\omega), \tag{7}$$

which act on function $\mu(\theta, \omega)$ in the usual way

$$(\gamma \cdot \mu)(\theta, \omega) = \mu(\gamma^{-1}(\theta, \omega)),\ \gamma \in O(2). \tag{8}$$

One notes that the evolution equation for $\mu(\theta, \omega)$ has $O(2)$ symmetry, provided that $g(\omega) = g(-\omega)$. More specifically, the equations are invariant under the actions of rotations and reflections, i.e., $\gamma \cdot (\mathscr{L} \cdot \mu) = \mathscr{L}(\gamma \cdot \mu)$ and $\gamma \cdot \mathscr{N}(\mu) = \mathscr{N}(\gamma \cdot \mu)$ for all $\gamma \in O(2)$.

The Fourier expansion for $\mu(\theta, \omega, t)$ is

$$\mu(\theta, \omega, t) = \sum_{l=-\infty}^{\infty} \mu_l(\omega, t) e^{il\theta}, \tag{9}$$

and the corresponding linear operator $\mathscr{L}$ can be rewritten as

$$\mathscr{L} \cdot \mu = \sum_{l=-\infty}^{\infty} (\mathscr{L}_l \mathscr{N}_l) e^{il\theta}, \tag{10}$$

where

$$\mathscr{L}_l \mu_l = -il(\omega - ilD)\mu_l + \frac{K|\omega|}{2}(\delta_{l,1} + \delta_{l,-1}) \cdot \int_{-\infty}^{\infty} d\omega' \cdot g(\omega')\, \mu_l(\omega'), \tag{11}$$

Note that the normalization condition for the density function implies $\mu_{l=0} = 0$.

An adjoint operator for $\mathscr{L}$ can be defined as

$$(A, \mathscr{L}B) \equiv (\mathscr{L}^{\dagger} A, B), \tag{12}$$

and the inner product is defined as

$$(A, B) \equiv \int_0^{2\pi} d\theta \int_{-\infty}^{\infty} d\omega A(\theta, \omega)^* B(\theta, \omega). \tag{13}$$

The definitions above yield

$$\mathscr{L}^{\dagger} \mu = \sum_{l=-\infty}^{\infty} (\mathscr{L}_l^{\dagger} \mathscr{N}_l) e^{il\theta}. \tag{14}$$

In term of the Fourier expansion

$$(\mathscr{L}_l^{\dagger}\, \mu_l(\omega)) = il(\omega + ilD)\mu_l(\omega) + \frac{g(\omega)K}{2}(\delta_{l,1} + \delta_{l,-1}) \cdot \int_{-\infty}^{\infty} d\omega' |\omega'|\, \mu_l(\omega'). \tag{15}$$

The eigenvalue equation for the linear operator $\mathscr{L}$ reads

$$\mathscr{L}\Psi = \lambda\Psi, \tag{16}$$

which could be treated in each Fourier subspace. We let

$$\Psi(\theta,\omega) = e^{il\theta}\psi(\omega). \tag{17}$$

Then Eq. (16) reads

$$\mathscr{L}_l\,\psi = \lambda\psi. \tag{18}$$

After scaling the eigenvalue $\lambda = -ilz$, Eq. (18) becomes

$$[(\omega - z) - ilD]\psi = -\frac{ilK|\omega|}{2}(\delta_{l,1}+\delta_{l,-1})\int_{-\infty}^{\infty} d\omega'\, g(\omega')\psi(\omega'). \tag{19}$$

From the analysis above, when $|l| \neq 1$, the eigenvalue solution of Eq. (19) corresponds to a continuous spectrum for $\mathscr{L}$. However, for the case of $|l| = 1$, Eq. (19) becomes

$$[(\omega - z) - ilD]\psi = -\frac{ilK|\omega|}{2}\int_{-\infty}^{\infty} d\omega'\, g(\omega')\psi(\omega'), \tag{20}$$

imposing the normalization condition for $\psi(\omega)$

$$\int_{-\infty}^{\infty} d\omega'\, g(\omega')\psi(\omega') = 1, \tag{21}$$

consistency between (20) and (21) requires z to be a root of the following special function

$$\Lambda_l(z) = 1 + \frac{ilK}{2}\int_{-\infty}^{\infty} d\omega'\,\frac{g(\omega')|\omega'|}{\omega' - z - ilD}, \tag{22}$$

i.e., the roots of Eq. (22) determine the eigenvalues. Recall that the projection operator $\hat{P}_\lambda$ onto the generalized eigenspace for an isolated eigenvalue $\lambda = -ilz_0$ is defined by a contour integral [37]

$$\hat{P}_\lambda \cdot A = \frac{1}{2\pi i}\oint_{\Gamma_0} d\lambda'(\lambda' - \mathscr{L})^{-1}A, \tag{23}$$

where Γ_0 is a small loop enclosing λ in a counter clockwise sense. The resolvent operator $(\lambda - \mathscr{L})^{-1}$ can be expressed as

$$(\lambda - \mathscr{L})^{-1}A = \sum_{-l=-\infty}^{\infty}(\lambda - \mathscr{L}_l)^{-1}A_l = \sum_{l=-\infty}^{\infty} R_l(\lambda)A_l(\omega), \tag{24}$$

where

$$(\lambda - \mathscr{L})A_l = [\lambda + il\omega + l^2D]A_l - \frac{K}{2}|\omega|(\delta_{l,1}+\delta_{l,-1})\int_{-\infty}^{\infty} d\omega' g(\omega')A_l(\omega'), \tag{25}$$

multiplying $(\lambda - \mathscr{L}_l)$ for both sides of Eq. (24), one obtains

$$\begin{aligned} A_l &= (\lambda - \mathscr{L})R_l(\lambda)A_l(\omega) \\ &= [\lambda + il\omega + l^2D]R_l(\lambda)A_l(\omega) - \frac{K}{2}|\omega|(\delta_{l,1}+\delta_{l,-1})\int_{-\infty}^{\infty} d\omega' g(\omega')R_l(\lambda)A_l(\omega'). \end{aligned} \tag{26}$$

Multiplying $[\lambda + il\omega + l^2 D]^{-1}$ for both sides of Eq. (26) and doing inner product with $g(\omega)$, we get

$$\int_{-\infty}^{\infty} d\omega' g(\omega') R_l(\lambda) A_l(\omega') = \frac{\int_{-\infty}^{\infty} \frac{A_l(\omega')g(\omega')}{\lambda + il\omega' + l^2 D} d\omega'}{1 - \frac{K}{2}(\delta_{l,1} + \delta_{l,-1}) \int_{-\infty}^{\infty} d\omega' \frac{g(\omega')}{\lambda + il\omega' + l^2 D}}. \tag{27}$$

Substituting (27) into the Eq. (25) we find that the projection could be simplified to

$$\hat{P}_\lambda \cdot A = \frac{e^{i\theta}}{2\pi i} \oint_{\Gamma_0} d\lambda' \, R_1(\lambda') A_1 + \frac{e^{-i\theta}}{2\pi i} \oint_{\Gamma_0} d\lambda' \, R_{-1}(\lambda') A_{-1}, \tag{28}$$

where

$$R_l(\lambda) \, A_l(\omega) = \frac{A_l(\omega)}{il\omega + l^2 D + \lambda} + \frac{K|\omega|(\delta_{l,1} + \delta_{l,-1})/2}{\Lambda_l(il\lambda)(il\omega + l^2 D + \lambda)} \int_{-\infty}^{\infty} d\omega' \frac{g(\omega') A_l(\omega')}{il\omega' + l^2 D + \lambda}. \tag{29}$$

A rigorous analysis of the nature of the eigenvalue should take the real and complex, the simple roots and the semi-simple ones into consideration respectively [37]. Since in the frequency-weighted model, a pair of conjugated complex and simple roots z_0 of $\Lambda_l(z)$ emerge near the critical coupling strength K_c , which means

$$\Lambda_1(z_0) = 0, \ \Lambda_1'(z_0) \neq 0. \tag{30}$$

Hence, we will focus our attention on this situation. From Eq. (20) $\sim$ Eq. (22) the eigenvalue $\lambda = -ilz_0$ has eigenvector $\Psi(\theta, \omega)$ and $\kappa \cdot \Psi(\theta, \omega)$, where

$$\Psi(\theta, \omega) = e^{i\theta} \psi(\omega) = e^{i\theta} \frac{-iK/2|\omega|}{\omega - z - iD}. \tag{31}$$

Meanwhile, its complex conjugate λ^* has the eigenvector $\Psi(\theta, \omega)^*$ and $\kappa \cdot \Psi(\theta, \omega)^*$, corresponding to Ψ and $\kappa \cdot \Psi$. There are adjoint eigenvectors

$$\mathscr{L}^\dagger \cdot \tilde{\Psi} = \lambda^* \tilde{\Psi}, \tag{32}$$

$$\mathscr{L}^\dagger (\kappa \cdot \tilde{\Psi}) = \lambda^* (\kappa \cdot \tilde{\Psi}), \tag{33}$$

where

$$\tilde{\Psi}(\theta, \omega) = \frac{e^{i\theta}}{2\pi} \frac{-g(\omega)}{\omega - z_0^* + iD} \frac{1}{\Lambda_1'(z_0)^*}. \tag{34}$$

These eigenvectors satisfy the orthogonal relations

$$(\tilde{\Psi}, \Psi) = (\kappa \cdot \tilde{\Psi}, \kappa \cdot \Psi) = 1, \tag{35}$$

$$(\tilde{\Psi}, \kappa \cdot \Psi) = (\kappa \cdot \tilde{\Psi}, \Psi) = 0. \tag{36}$$

Furthermore, the projection $\hat{P}_\lambda$ in Eq. (28) can be evaluated through residue theorem and the final result has the simplified form

$$\hat{P}_\lambda \cdot \mu = (\tilde{\Psi}, \mu) \Psi + (\kappa \cdot \tilde{\Psi}, \mu) \kappa \cdot \Psi. \tag{37}$$

Defining the amplitudes (α, β)

$$\alpha(t) = (\tilde{\Psi}, \mu), \tag{38}$$

$$\beta(t) = (\kappa \cdot \tilde{\Psi}, \mu), \tag{39}$$

the distribution function $\mu(\theta, \omega, t)$ decomposes as

$$\begin{aligned} \mu(\theta, \omega, t) &= \hat{P_\lambda} \cdot \mu + \hat{P_{\lambda^*}} \cdot \mu + S(\theta, \omega, t) \\ &= [\alpha(t)\Psi(\theta, \omega) + \beta(t)\kappa \cdot \Psi(\theta, \omega) + c.c.] + S(\theta, \omega, t), \end{aligned} \tag{40}$$

where $S(\theta, \omega, t)$ is the remaining degree of freedom and $(\tilde{\Psi}, S) = (\kappa \cdot \tilde{\Psi}, S) = 0$. Inserting Eq. (40) into the perturbed equation Eq.(35) (in the main text) and projecting with Ψ and $\kappa \cdot \tilde{\Psi}$, we can obtain the following equation in terms of the Fourier exponents [37]

$$\frac{d\alpha}{dt} = \lambda\alpha - \pi K(\alpha^* + \beta + \langle g, S_{-1}\rangle)\langle \tilde{\psi}, S_2\rangle, \tag{41}$$

$$\frac{d\beta}{dt} = \lambda\beta - \pi K(\alpha + \beta^* + \langle g, S_1\rangle)\langle \kappa \cdot \tilde{\psi}, S_{-2}\rangle, \tag{42}$$

$$\begin{aligned} \frac{\partial S}{\partial t} =& \mathscr{L} S - \pi K\{e^{i\theta}(\alpha^* + \beta + \langle g, S_{-1}\rangle)[S_2 - \langle \tilde{\psi}, S_2\rangle \psi \\ & - \langle \kappa \cdot \tilde{\psi}^*, S_2\rangle \kappa \cdot \psi^*] + 2e^{i2\theta}[(\alpha^* + \beta + \langle g, S_{-1}\rangle)S_3(\omega) \\ & - (\alpha + \beta^* + \langle g, S_1\rangle)(\alpha\psi + \beta^*\kappa \cdot \psi^* + S_1)] + \\ & \sum_{l \geq 3} l e^{il\theta}[(\alpha^* + \beta + \langle g, S_{-1}\rangle)S_{l+1}(\omega) - (\alpha + \beta^* + \\ & \langle g, S_1\rangle)S_{l-1}(\omega)] + c.c.\}, \end{aligned} \tag{43}$$

where $\langle A, B\rangle = \int_{-\infty}^{\infty} d\omega\, A^* B$. For a solution on the center manifold near the incoherent state, the time dependence of $S(\theta, \omega, t)$ can be expressed as

$$\begin{aligned} S(\theta, \omega, t) &= H(\theta, \omega, \alpha(t), \alpha(t)^*, \beta(t), \beta(t)^*) \\ &= \sum_{l=-\infty}^{\infty} H_l(\omega, \alpha(t), \alpha(t)^*, \beta(t), \beta(t)^*)e^{il\theta}. \end{aligned} \tag{44}$$

Besides, if $\mu(\theta, \omega)$ corresponds to a point on the center manifold and $\gamma \in O(2)$, then $\gamma \cdot \mu$ still lies on the center manifold. This symmetry imposes the constraints on the form of H. The density function $\mu(\theta, \omega, t)$ can be rewritten as

$$\begin{aligned} \mu(\theta, \omega, t) =& \alpha\Psi(\theta, \omega) + \beta\kappa \cdot \Psi(\theta, \omega) + \alpha^*\Psi(\theta, \omega)^* + \\ & \beta^*\kappa \cdot \Psi(\theta, \omega)^* + H(\theta, \omega, \alpha(t), \alpha^*(t), \beta(t), \beta^*(t)). \end{aligned} \tag{45}$$

Because of the $O(2)$ symmetry, any action of $\gamma \in O(2)$ on $\mu(\theta, \omega, t)$ keeps the same form as (45), i.e.,

$$\begin{aligned} \gamma \cdot \mu(\theta, \omega, t) =& \alpha'\Psi(\theta, \omega) + \beta'\kappa \cdot \Psi(\theta, \omega) + \alpha^{*'}\Psi(\theta, \omega)^* + \\ & \beta^{*'}\kappa \cdot \Psi(\theta, \omega)^* + H(\theta, \omega, \alpha'(t), \alpha^{*'}(t), \beta'(t), \beta^{*'}(t)). \end{aligned} \tag{46}$$

Following the definition, the mode amplitude transforms according to

$$\phi \cdot (\alpha, \beta) = (e^{-i\phi}\alpha, e^{i\phi}\beta), \tag{47}$$

$$\kappa \cdot (\alpha, \beta) = (\beta, \alpha). \tag{48}$$

Hence, $H(\theta, \omega, \alpha(t), \alpha^*(t), \beta(t), \beta^*(t))$ must satisfy

$$H(\theta - \phi, \omega, \alpha, \alpha^*, \beta, \beta^*) = H(\theta, \omega, e^{-i\phi}\alpha, e^{i\phi}\alpha^*, e^{i\phi}\beta, e^{-i\phi}\beta^*), \tag{49}$$

and

$$H(-\theta, -\omega, \alpha, \alpha^*, \beta, \beta^*) = H(\theta, \omega, \beta, \beta^*, \alpha, \alpha^*). \tag{50}$$

The Fourier coefficient of H satisfies

$$e^{il\phi}H_l(\omega, \alpha, \alpha^*, \beta, \beta^*) = H_l(\omega, e^{-i\phi}\alpha, e^{i\phi}\alpha^*, e^{i\phi}\beta, e^{-i\phi}\beta^*), \tag{51}$$

and

$$H_l(-\omega, \alpha, \alpha^*, \beta, \beta^*) = H_l(\omega, \beta, \beta^*, \alpha, \alpha^*). \tag{52}$$

The constraint (51) implies that the function $(\alpha^*)^l H_l$ is invariant under rotations, so it can only depend on the basic rotation invariants $|\alpha|^2$, $|\beta|^2$, $\alpha\beta$, and $\alpha^*\beta^*$. We assume $(\alpha^*)^l H_l$ takes the form

$$(\alpha^*)^l H_l = \sum_{j=0}^{l} |\alpha|^{2j}(\alpha^*\beta^*)^{l-j} H_l^{(j)}(\omega, |\alpha|^2, |\beta|^2, \alpha\beta, \alpha^*\beta^*), \tag{53}$$

The Fourier coefficients of H have the form

$$H_l = \sum_{j=0}^{l} \alpha^l (\beta^*)^{l-j} H_l^{(j)}(\omega, |\alpha|^2, |\beta|^2, \alpha\beta, \alpha^*\beta^*), \tag{54}$$

for $l > 0$. Hence $H_l^{(j)}$ are functions of $|\alpha|^2, |\beta|^2, \alpha\beta, \alpha^*\beta^*$, and ω that satisfy

$$H_l^{(j)}(-\omega, |\alpha|^2, |\beta|^2, \alpha\beta, \alpha^*\beta^*)^* = H_l^{(l-j)}(\omega, |\alpha|^2, |\beta|^2, \alpha\beta, \alpha^*\beta^*). \tag{55}$$

For $l = 1$ and $l = 2$, in terms of the components in Eq. (54) and Eq. (55), we have

$$H_1 = \alpha H_1^{(1)} + \beta^* H_1^{(0)}, \tag{56}$$

$$H_2 = \alpha^2 H_2^{(2)} + \alpha\beta^* H_2^{(1)} + (\beta^*)^2 H_2^{(0)}, \tag{57}$$

Near the critical point the amplitude is small, so H could be expressed in terms of the Taylor series. Since the center-manifold geometry requires that the Taylor expansion of H begins at second order, the leading term of H is

$$H_1 = \mathscr{O}(3), \tag{58}$$

$$H_2 = \alpha^2 h_2^{(2)}(\omega) + \alpha\beta^* h_2^{(1)}(\omega) + (\beta^*)^2 h_2^{(0)}(\omega) + \mathscr{O}(3). \tag{59}$$

The high-order terms could be neglected to determine the cubic term in the amplitude equation. The four-dimensional vector field Eq. (41) $\sim$ Eq. (42) could be transformed to the Poincare-Birkhoff normal form which introduces a "phase shift invariant" characterizing the Hopf normal form. In the normal form the four-dimensional equation reads

$$\begin{pmatrix} \dot{\alpha} \\ \dot{\beta} \end{pmatrix} = \lambda \begin{pmatrix} \alpha \\ \beta \end{pmatrix} - \pi K \begin{pmatrix} \alpha[\langle \tilde{\psi}, h_2^{(2)} \rangle |\alpha|^2 + \langle \tilde{\psi}, h_2^{(1)} \rangle |\beta|^2] \\ \beta[\langle \kappa \cdot \tilde{\psi}, h_2^{(0)*} \rangle |\beta|^2 + \langle \kappa \cdot \tilde{\psi}, h_2^{(1)*} \rangle |\alpha|^2] \end{pmatrix} + \mathscr{O}(5). \tag{60}$$

Let $|\alpha|^2 = \dfrac{(u-\delta)}{2}$ and $|\beta|^2 = \dfrac{(u+\delta)}{2}$, Eq. (60) becomes

$$\begin{aligned} \begin{pmatrix} \dot{\alpha} \\ \dot{\beta} \end{pmatrix} =& [\lambda - \frac{\pi K}{2}(\langle \tilde{\psi}, h_2^{(2)} \rangle + \langle \tilde{\psi}, h_2^{(1)} \rangle) u + \mathscr{O}(4)] \begin{pmatrix} \alpha \\ \beta \end{pmatrix} \\ &+ [\frac{\pi K}{2}(\langle \tilde{\psi}, h_2^{(2)} \rangle - \langle \tilde{\psi}, h_2^{(1)} \rangle) + \mathscr{O}(2)] \delta \begin{pmatrix} \alpha \\ -\beta \end{pmatrix}, \end{aligned} \tag{61}$$

where $h_2^{(1)}$, $h_2^{(2)}$ can be determined consistently from the evolution of Eq. (43) based on the balance equation of α. After similar calculations we obtain

$$h_2^{(2)}(\omega) = \frac{-i\pi K |\omega| \psi(\omega)}{\omega - z_0 - i2D}, \tag{62}$$

$$h_2^{(1)}(\omega) = \frac{-i\pi K |\omega| (\psi(\omega) + \kappa \cdot \psi(\omega)^*)}{\omega - i\mathrm{Im} z_0 - i2D}, \tag{63}$$

and the cubic coefficients P_u and r read

$$P_u(0) = \frac{\pi K}{2} \lim_{D \to 0^+} \mathrm{Re} \langle \tilde{\psi}, (h_2^{(1)} + h_2^{(2)}) \rangle, \tag{64}$$

$$r(0) = \frac{-\pi K}{2} \lim_{D \to 0^+} \mathrm{Re} \langle \tilde{\psi}, (h_2^{(1)} - h_2^{(2)}) \rangle. \tag{65}$$

Finally, the stability and direction of the bifurcation for the traveling wave and standing wave are determined by the cubic coefficients P_u and r. Ref. [37] provided the detailed bifurcation diagram for Hopf bifurcations in the $P_u - r$ parameter plane. Totally, there are six different types of bifurcation mechanisms.
